# Model systems to elucidate minimum requirements for protected areas networks

**DOI:** 10.1038/s41598-019-56142-2

**Published:** 2019-12-20

**Authors:** Yolanda F. Wiersma, R. Troy McMullin, Darren J. H. Sleep

**Affiliations:** 1 Department of Biology, Memorial University, St. John’s, NL Canada; 2 Resarch and Collections, Canadian Museum of Nature, Ottawa, ON Canada; 3 Conservation Strategies, Sustainable Forestry Initiative Inc., Ottawa, ON Canada

**Keywords:** Biodiversity, Conservation biology

## Abstract

In conservation biology there have been varying answers to the question of “How much land to protect?” Simulation models using decision-support software such as Marxan show that the answer is sensitive to target type and amount, and issues of scale. We used a novel model system for landscape ecology to test empirically whether the minimum conservation requirements to represent all species at least once are consistent across replicate landscapes, and if not, whether these minimum conservation requirements are linked to biodiversity patterns. Our model system of replicated microcosms could be scaled to larger systems once patterns and mechanisms are better understood. We found that the minimum representation requirements for lichen species along the microlandscapes of tree trunks were remarkably consistent (4–6 planning units) across 24 balsam fir trees in a single stand, as well as for 21 more widely dispersed fir and yellow birch trees. Variation in minimum number of planning units required correlated positively with gamma diversity. Our results demonstrate that model landscapes are useful to determine whether minimum representation requirements are consistent across different landscapes, as well as what factors (life history, diversity patterns, dispersal strategies) affect variation in these conservation requirements. This system holds promise for further investigation into factors that should be considered when developing conservation designs, thus yielding scientifically-defensible requirements that can be applied more broadly.

## Introduction

“How much to protect” has been a pressing question for conservation biologists in both the academic and practitioner literature^1^. Research questions related to systematic conservation planning (SCP) have focused on evaluations of how different target types (such as percentage of land area or proportion of populations)^2,3^ and amounts (e.g., 12% vs. 50% of land area)^4–6^, effects of scale^7,8^, planning unit size^9^ and data characteristics^10,11^ affect conservation planning outcomes. For example, researchers have examined how the scale at which planning takes place affects the number and location of protected areas needed to capture a representative proportion of biodiversity^7–9^. A critical question for conservation planners is whether there are guidelines for a fixed percentage of land area that should be set aside for conservation that can ensure that all species are adequately represented^1,2^. Such “percentage targets” are appealing for governments and conservation organizations in that they are simple to understand and measure, and provide a tool to evaluate how well agencies are moving towards set goals. However, whether published targets of 12%^12^ or 50%^13^ are sufficient to adequately represent and protect biodiversity is not known. The vast number of research papers that have addressed this question (see reviews at refs. ^3,14,15^ for details) have failed to come up with a consistent answer to the question of “how much to protect?”; consensus in the form of multi-authored position papers^13^ appear to be based more on normative claims than empirical evidence.

This lack of consistent estimates for the percentage of land necessary to meet particular conservation requirements (for example, how much land needs to be set aside to capture all species in a protected area at least once) may be attributable to differences in the respective ecological systems. Minimum representation requirements will be different in a highly biodiverse tropical rainforest than a less diverse high latitude system. Differences in spatial heterogeneity, both of abiotic and biotic patterns will also have an effect on conservation targets^10,16^. These *prima facie* differences across studies may explain much of the observed lack of consistency in recommended percentage targets for effective conservation, but these differences also overlook the one limitation that all SCP studies hold in common; the difficulty to rigorously test questions about different aspects of conservation design decisions through experimentation. While SCP exercises are not generally considered “experimental”, SCP is viewed as a tool to make decision making scientific rigorous and transparent^17^. The strength of SCP as a decision-support tool^1^ is through the use of models of one type or another (e.g., heuristic models, optimization models). Models are simply expressions of hypotheses given rise, in the case of SCP, from questions generally along the lines of “is a design criterion of X sufficient to conserve biological features Y in area Z?” Within SCP, the design criteria can include issues about the size of individual planning units (candidate protected areas), the type of target (e.g., minimum number of sites, minimum percentage area) and the target amount (e.g., 12%, 50%).

The hallmarks of experimentation (whether manipulative or observational) are controls, randomization, and replication. Considering conservation planning examples as experiments that can help to develop general guidelines for conservation targets thus faces a challenge. The experimental units for SCP are usually biologically- or politically-bounded regions that are hundreds to thousands of kilometers in extent. These cannot feasibly be replicated. Adjacent bioregions or provinces/states are not suitable replicate experimental units because, due to large spatial scales of landscapes, they are too different to be able to attribute differences in outcomes to the “treatment” (which could, for example, be different decisions about conservation design – such as number or size of protected areas) vs. underlying differences in the study areas themselves.

Researchers have tried to get around the problem of replication through *in silico* analyses. Conservation planning software (the most commonly applied of which is Marxan^15,18^) allows researchers to conduct thousands to tens of thousands of “runs” of conservation scenarios to examine how different inputs/constraints affect the conservation output (for an example see ref. ^1^) and as a form of sensitivity analysis. Other software algorithms (e.g., Zonation^19^) use slightly different approaches; however all *in silico* analyses are constrained within the same region and on the same data, and hence they are not replicates. Despite that experimentation with replicates at landscape is impossible, it does behoove conservation biologists to consider whether there are alternative ways to increase the empirical rigour of SCP. Increased rigour would help conservation biologists to determine whether conservation targets are situation-specific and not the kind for which generalizations can be made. We propose the use of a novel model system to determine whether consistent “rules” for minimum conservation requirements can be developed.

Model systems have been widely used in biomedical science (i.e., *Rattus norvegicus*, *Drosophola melanogaster*). Model systems in biomedical research are necessary to address the ethical and logistical challenges of doing medical experiments on humans. Fruit flies and small animals such as mice, rats and zebrafish are easy to maintain in laboratory environments and respond quickly to experimental treatments. This enables manipulative experiments with statistically relevant sample sizes. The genetics and physiology of lab organisms are considered similar enough to that of humans that we can extrapolate findings from these organisms to decisions about how to treat complex diseases in humans, such as cancer^20^ and Parkinson’s^21^.

**Figure 1:**
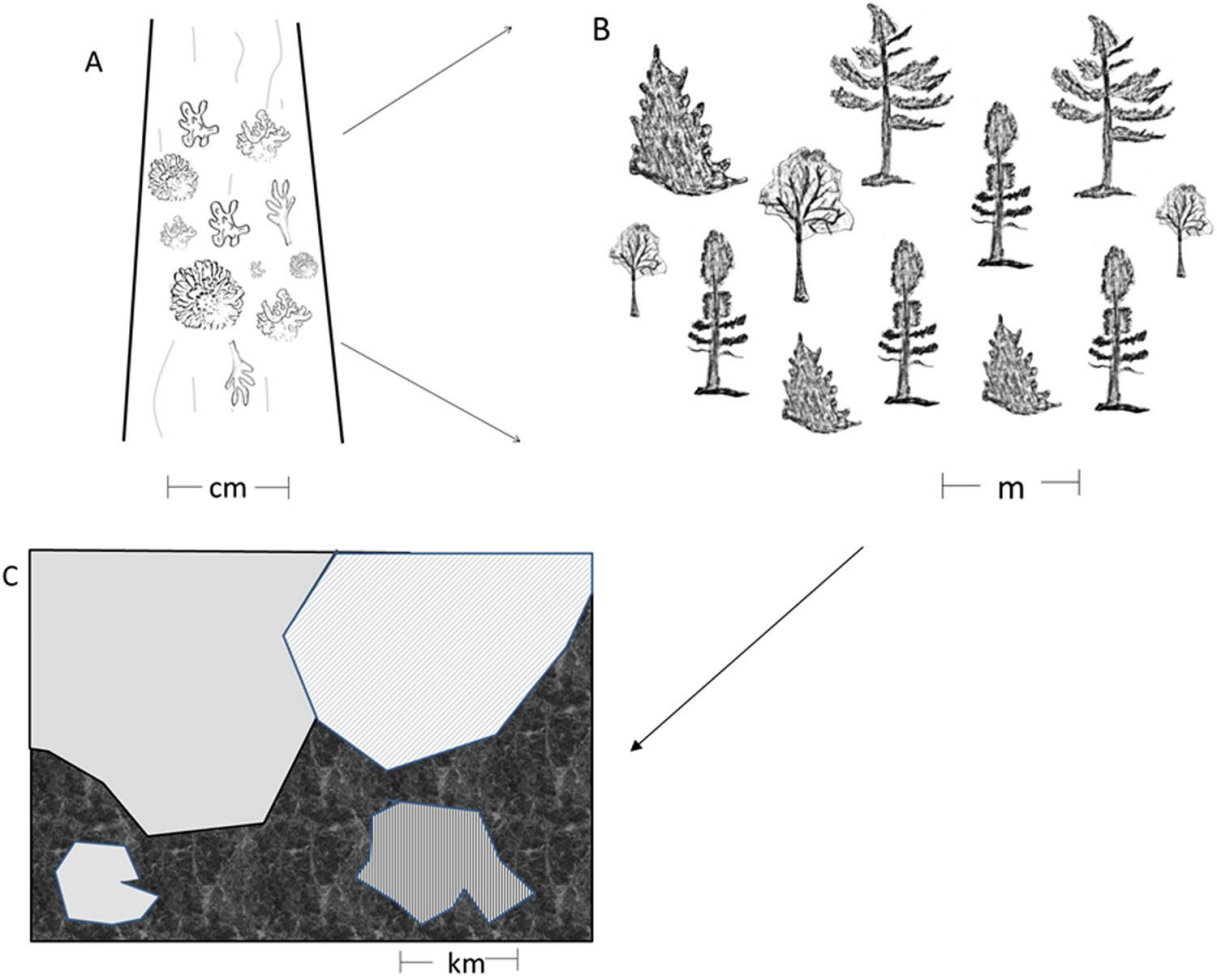
A schematic of the hierarchical structure of the proposed model system for landscapes. At the smallest scale (panel A) the landscape is centimeters in extent and is comprised of patches of lichens on the bole for trees. Each tree can be considered a replicate microlandscape. Within a single forest stand (panel B), the landscape is metres in extent. Some stands are homogenous in terms of tree species, age/size, but across the stand there will be fine-grained heterogeneity in microtopography and microclimate. These stands are then patches in a kilometers-extent landscape (panel C); some stands of similar composition may be replicated across the landscape but interspersed with other patch types (e.g., non-forested bog, meadow, anthropogenic patches).

The use of model systems has been encouraged in ecology^22^. Microcosms^23^ act as model systems for a wide array of ecological questions in population and community ecology. However existing microcosms such as patches of mosses^24^ or bromeliads^25^ are binary systems of habitat/non-habitat which makes them less realistic as model systems for landscape ecology. Model systems for landscape ecology, which are characterized by a patch-mosaic structure or a gradient of features, instead of a binary one, are less common, although there have been some proposed systems. For example, biocrusts^26^ and lichen covered trunks of trees of the same species growing in the same stand have been proposed as model systems for landscapes^27^. In this latter model system, the patterns of lichen species occurrence along the trunks of trees were shown to be statistically consistent across 24 trees sampled in a single stand and across a broader region for lichens living on two different species of trees^27^. Where certain species of lichens were located along a 1 m gradient of the trunk was statistically similar across multiple trees, much the same way that vegetation patterns along an elevation gradient follow predictable transitions from temperate to coniferous forest to alpine plants. Thus, we can consider lichen thalli as analogous to patches of different land cover types, and individual trees are analogues to a landscape^27,28^, making replicate experimental landscapes (i.e., with replicate trees) possible (Fig. 1). In this study, we harness this model system to experimentally test a key question in SCP, that of whether a fixed number of planning units can be uniformly applied across different landscapes to achieve the same conservation outcome of having all species in the system represented in at least one protected area.

## Results

**Figure 2:**
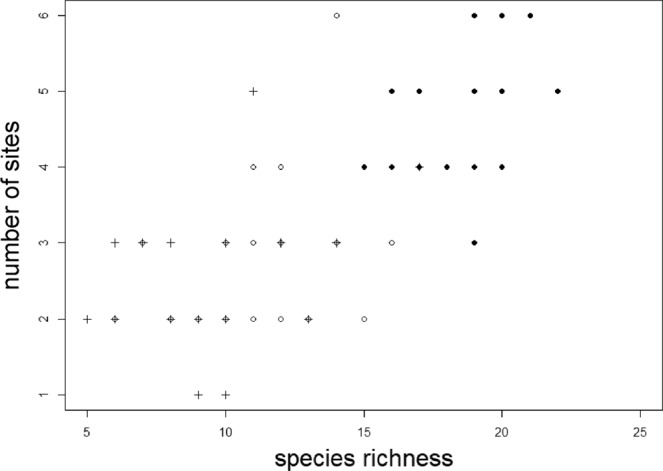
Number of sites (10 cm × 10 cm “planning units”) required to represent all lichens on model systems of landscapes as a function of species richness. Trees were all growing within the Avalon Forest Ecoregion, Newfoundland, Canada, and include 24 balsam fir trees (*Abies balsamea*) growing in a single stand (black circles) and 21 growing across the wider ecoregion (open circles), along with 21 yellow birch (*Betula allegheniensis*) growing across the wider ecoregion (plus (+) symbols), an area of ~500 km^2^.

At the scale of individual trees (each acting as an individual model landscape, each subdivided into 20 smaller “planning units”) within a single stand, we found that 3–6 planning units per tree were needed to represent all lichen species on each tree at least once (Fig. 2; Table 1). When applied to replicate trees at the wider ecoregion, between 2–6 planning units per tree were needed to represent all lichen species at least once on 21 balsam fir trees and between 1–5 planning units per tree were needed to represent all lichen species on 21 yellow birch trees (Fig. 2; Table 1).

**Table 1:** Summary of minimum requirements to represent lichens at three sampling extents (first column) on trees in the Avalon Forest Ecoregion on the island of Newfoundland.

Sampling extent	Mean (±sd) species Richness	Mean (±sd) number of planning units per tree	Total species richness in across all trees	Number of trees to represent sampling extent
24 balsam fir (single stand)	18.75 (1.66)	4.76 (0.85)	37	5
21 balsam fir (ecoregion)	11.24 (2.60)	2.86 (1.04)	27	6
21 yellow birch (ecoregion)	9.29 (2.88)	2.52 (0.96)	31	6

In the first case, we treat trees as the experimental replicate, which have varying species richness (second column) and we ask how many “planning units” (10 cm × 10 cm sampling blocks) are required to represent all species on each tree (third column). In the second case, we treat the stand/region as the experimental unit (no true replication) and examine how many individual trees (last column) are required to represent the gamma diversity (fourth column) in the stand/region.

**Figure 3:**
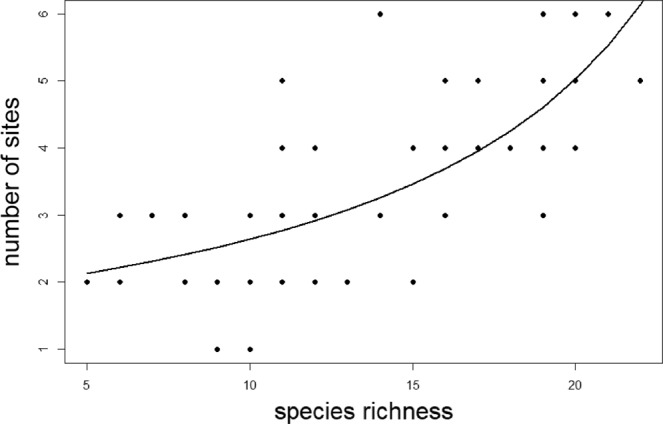
Number of sites (10 cm × 10 cm “planning units”) required to represent all lichens on model systems of landscapes (individual trees, *n* = 66) as a function of species richness. Trees were all growing within the Avalon Forest Ecoregion, Newfoundland, Canada, and include 24 balsam fir trees (*Abies balsamea*) growing in a single stand and 21 growing across the wider ecoregion, along with 21 yellow birch (*Betula allegheniensis*) growing across the wider ecoregion, an area of ~500 km^2^. The data on the y-axis is the number of sites identified using the Simulated Annealing with Gradual Improvement option and 1000 iterations in Marxan. This value ranged from 2–6 (see Table 1 and Results section for details) to represent all species at least once. Line of fit is a GLM with a Gamma function, R^2^ = 0.4898, p = 2.78e-11.

At the extent of the stand, the best (most effective and efficient) scenario identified that five planning units (i.e., 5 trees) were required to represent all 37 lichen species in the stand at least once (Table 1). At the extent of the ecoregion, the best scenario identified that for balsam fir, six planning units (i.e., 6 trees) were required to represent all 27 lichen species at least once (Table 1), and that for yellow birch, six planning units (i.e., 6 trees) were required to represent all 31 lichen species at least once (Table 1). Across all three sets of runs using trees as model landscapes there was a statistically significant trend (R^2^ = 0.4898, p = 2.78e-11) of trees with higher lichen diversity requiring more sites to represent all species (Fig. 3).

## Discussion

Although a protected area strategy, even for small, non-mobile species such as lichens, would not be advisable at the extent of centimetres or metres, this analysis has value in that it allows for tests of consistency in conservation designs within a system of statistically similar micro-landscapes. When we had true experimental replicates, the number of planning units required to represent all species fell consistently between 2–6 for the lichen communities living on the balsam fir and between 1–5 for the lichen communities on yellow birch.

What factors influence this observed variation in the number of sites necessary to represent lichen biodiversity is unknown. It would be important to understand drivers of conservation requirements in this model system to extrapolate these findings and develop scientifically defensible conservation targets at kilometres-extent landscapes. Species diversity (gamma diversity) can be an important driver – with areas that have higher diversity predicted to require higher percentages of land set aside for conservation. In this model system, gamma diversity explains 49% of the variation in the number of sites required to represent all species (Fig. 3). Thus, we posit that gamma diversity at kilometres-extent landscapes might be an important driver of how much land needs to be set aside as protected in a real-world SCP exercise.

Other factors that might be important for determining protected areas requirements include beta diversity^29^, ecosystem productivity^30^, population dynamics^31^ and species-area relationships^11^. The advantage of the model system here is that the spatial extent of study areas (sizes of individual trees) along with abiotic drivers (climate, soils) are held relatively constant, while species diversity on each tree varies. The value of using a model system lies in the ability to have experimental replicates to test hypotheses about conservation requirements in a way that is not possible in larger systems. Because ecological systems are hierarchical^32^ we believe that patterns from the model systems (as shown in Fig. 1) can be extrapolated out to real-world kilometers-extent landscapes with similar levels of alpha, beta or gamma diversity of appropriately scaled taxa. Based on the strength of experimental replication, conservation planners could be reasonably confident that a target based on an empirical relationship to diversity (Fig. 3) could achieve minimum representation requirements.

A recent global survey of conservation planning initiatives found that very few plans included rigorous evaluations of effectiveness^33^. This is not to say that conservation initiatives are not effective at conserving biodiversity, but rather that resources are not invested in rigorous analysis. The use of a model system such as this might be a cost-effective means to take a “first look” at how variation in conservation planning elements affects outcomes. In addition to helping to guide conservation planning at real-world extents, model systems allow for increased rigour and experimentation to test hypotheses about whether and how variation in diversity patterns influences conservation design. Such experiments may help conservation biologists to make scientifically defensible decisions on both *how much* to protect, but also explain *why* that amount is sufficient.

## Methods

### Study system

The study area is the Central Avalon Forest Ecoregion, on the island of Newfoundland, Canada. The area (500 km^2^) is a globally recognized lichen biodiversity hotspot^34^ with a climate characterized by cool, moist summers and mild winters. Land cover is mainly forests dominated by balsam fir (*Abies balsamea*), which occur along the slopes and tops of ribbed moraines — glacial features of rolling hills^35^ — interspersed with sphagnum-dominated bogs^36^. Along with the balsam fir trees, black spruce (*Picea mariana*) can be found in wet areas and yellow birch (*Betula alleghaniensis*) occur sporadically throughout the forest, mostly on upland areas.

We replicate our test for whether SCP have a consistent outcome in terms of the minimum number of sites required to represent all species at least once using a model system with two different sampling schema. In the first sampling scheme, we sampled 24 balsam fir trees within a single transect (100 m × 5 m). Trees were of a similar age and we sampled them for macro-lichen diversity by placing a 1 m × 0.1 m “microtransect” vertically along the north- and south-facing sides of the tree bole (starting 0.9 m from the ground), and counting the species richness and number of thalli within 10 cm sample blocks along the transect. Trees contained between 15–22 lichen species each, and overall (gamma) diversity of lichens in the stand was 37. The individual 10 cm × 10 cm blocks contained between 3–12 species and are the analog to “planning units” in the SCP framework. Details on the larger ecoregion characteristics and the lichen sampling and identification protocol can be found in ref. ^27^. Thus, this first scheme is analogous to a conservation plan occurring across 24 replicate landscapes (i.e., statistically similar landscapes) each of which contain 15–22 species (conservation targets). Within each replicate landscape, there are 20 potential planning units (ten 10 cm × 10 cm plots along both the north and south sides of the trunk), from which we selected the minimum set that represents all species via the Marxan algorithm (described below). The number of planning units per tree is constrained by logistics and sampling protocols described in ref. ^27^.

In the second sampling scheme, we sampled 21 sites dispersed across the ecoregion. At each site, we inventoried the diversity of lichens on one balsam fir and one yellow birch (21 trees of each species total). We used a similar microtransect composed of 10 cm × 10 cm sample blocks to sample lichen diversity on both the north and south sides of each tree, but this transect was only 0.5 m long and started at 1.1 m from the ground and sampled for both macro- and micro-lichens. Thus, in the second sampling scheme there were half as many planning units as in the first, but a larger suite of potential species sampled in each, and there were 21 replicate “landscapes” each for balsam fir and yellow birch. Balsam fir trees contained between 6–16 lichen species (gamma diversity across all 21 fir trees was 27) and yellow birch trees contained between 5–17 lichen species (gamma diversity across all 21 birch trees was 31). The individual 10 cm × 10 cm blocks contained between 1–12 species on balsam fir and between 0–11 species on yellow birch. Details on the lichen sampling and identification protocol can be found in ref. ^28^.

### Marxan analysis

We used lichen data from both sampling schema to carry out a systematic conservation planning (SCP) exercise at two spatial extents. At the first extent, we treated trees as model systems for landscapes. Within each tree, we used the 10 cm × 10 cm sampling blocks as the planning units and asked how many of these would need to be set aside to represent all species of lichens on that tree at least once. In this case, we had 24 experimental replicates in the first sampling scheme and 21 experimental replicates each of two tree species in the second sampling scheme. At the larger extent (stand and ecoregion), we set each individual tree as the planning unit and asked how many trees needed to be set aside to represent each lichen species in the stand or ecoregion at least once.

In all cases, we applied Marxan^18^ using the Simulated Annealing with Gradual Improvement option and 1000 iterations. Cost of planning units was set to 1 (default) in all cases, making planning unit costs invariant. For the tree-level planning scenario, we examined the relationship between the number of 10 cm × 10 cm blocks required to represent all species on a tree and species richness, across all 66 trees using a GLM (Gamma family, identity link) in the statistical software R^37^.

## Data Availability

The lichen data used for this analysis are available from the corresponding author upon request. Upon acceptance, we will publish the data in an online repository (e.g., Dryad, FigShare). The lichen data used for this analysis are published on FigShare at https://figshare.com/articles/Lichen_Data/11310077.

## References

[1] Margules CR, Pressey RL (2000). Systematic conservation planning. Nature.

[2] Tear TH (2005). How much is enough? The recurrent problem of setting measurable objectives in conservation. Bioscience.

[3] Kukkala AS, Moilanen A (2013). Core concepts of spatial prioritisation in systematic conservation planning. Biol. Rev..

[4] Solomon M, Van Jaarsveld AS, Biggs HC, Knight MH (2003). Conservation targets for viable species assemblages?. Biodivers. Conserv..

[5] Svancara LK (2005). Policy-driven versus evidence-based conservation: A review of political targets and biological needs. Bioscience.

[6] Wiersma YF, Nudds TD (2006). Conservation targets for viable species assemblages in Canada: are percentage targets appropriate?. Biodivers. Conserv..

[7] Wiersma YF (2007). The effect of target extent on the location of optimal protected areas networks in Canada. Landsc. Ecol..

[8] Justus J, Fuller T, Sarkar S (2008). Influence of representation targets on the total area of conservation-area networks. Conserv. Biol..

[9] Rouget M (2003). Measuring conservation value at fine and broad scales: implications for a diverse and fragmented region, the Agulhas Plain. Biol. Conserv..

[10] Kujala H, Moilanen A, Gordon A (2018). Spatial characteristics of species distributions as drivers in conservation prioritization. Methods Ecol. Evol..

[11] Drira S, Ben Rais Lasram F, Ben Rejeb Jenhani A, Shin YJ, Guilhaumon F (2019). Species-area uncertainties impact the setting of habitat conservation targets and propagate across conservation solutions. Biol. Conserv..

[12] McNeely, J. & Miller, K. (eds). *National Parks Conservation and Development: the Role of Protected Areas in Sustaining Society*, *Proceedings of the World Congress on National Parks*. (Smithsonian Institution Press, 1984).

[13] Dinerstein E (2017). An ecoregion-based approach to protecting half the terrestrial realm. BioScience.

[14] Pressey RL, Cabeza M, Watts ME, Cowling RM, Wilson KA (2007). Conservation planning in a changing world. Trends Ecol. Evol..

[15] Wiersma YF, Sleep DJH (2016). A review of applications of the six-step method of systematic conservation planning. For. Chron..

[16] Wang J (2018). Spatial relationship between climatic diversity and biodiversity conservation value. Conserv. Biol..

[17] Schwartz MW (2018). Decision support frameworks and tools for conservation. Cons. Lett..

[18] Ball, I. R., Possingham, H. P. & Watts, M. E. *Marxan and Relatives: Software for Spatial Conservation Prioritization*. *Spatial conservation prioritisation: quantitative methods and computational tools*. (Oxford University Press, 2009).

[19] Moilanen Atte (2007). Landscape Zonation, benefit functions and target-based planning: Unifying reserve selection strategies. Biological Conservation.

[20] Tudrej P, Kujawa KA, Cortez AJ, Lisowska KM (2019). Characteristics of *in vivo* model systems for ovarian cancer studies. Diagnostics.

[21] Lu B, Vogel H (2009). Drosophila models of neurodegenerative diseases. Science.

[22] Vitousek PM (2002). Oceanic islands as model systems for ecological studies. J. Biogeogr..

[23] Srivastava DS (2004). Are natural microcosms useful model systems for ecology?. Trends Ecol. Evol..

[24] Gonzalez A, Lawton JH, Gilbert FS, Blackburn TM, Evans-Freke I (1998). Metapopulation dynamics, abundance, and distribution in a microecosystem. Science.

[25] Talaga S (2015). Tank bromeliads as natural microcosms: a facultative association with ants influences the aquatic invertebrate community structure. Comptes Rendus - Biol..

[26] Bowker MA (2014). Biological soil crusts (biocrusts) as a model system in community, landscape and ecosystem ecology. Biodivers. Conserv..

[27] Wiersma YF, McMullin RT (2018). Is it common to be rare on the landscape? A test using a novel model system. Landsc. Ecol..

[28] Wiersma, Y. F., Wigle, R. D. & McMullin, R. T. A proposed microcosm for landscape ecology – beyond the binary to the patch-mosaic model. *bioRxiv* **542985**, 10.1101/542985 (2019).

[29] Socolar JB, Gilroy JJ, Kunin WE, Edwards DP (2016). How should beta-diversity inform biodiversity conservation?. Trends Ecol. Evol..

[30] Andrew ME, Wulder MA, Coops NC (2011). Patterns of protection and threats along productivity gradients in Canada. Biol. Conserv..

[31] Rondinini C, Chiozza F (2010). Quantitative methods for defining percentage area targets for habitat types in conservation planning. Biol. Conserv..

[32] Wiens, J. A. Spatial scale and temporal variation in studies of shrubsteppe birds in *Community Ecology* (eds. Diamond, J & Case T. J.) 154–172 (Harper & Row, New York, 1986).

[33] McIntosh EJ (2018). Absence of evidence for the conservation outcomes of systematic conservation planning around the globe: a systematic map. Environmental Evidence.

[34] Ahti, T. Lichens. In *Biography and Ecology of the Island of Newfoundland*. *Monographiae* Biologicae *48*. (ed. South, G. R.) 319–360 (Dr. W. Junk Publishers, 1983).

[35] Hättestrand C, Kleman J (1999). Ribbed moraine formation. Quat. Sci. Rev..

[36] South, G. *Biogeography and Ecology of the Island of Newfoundland*. (Dr. W. Junk Publishers, The Hague, 1983).

[37] R Core Team. R: A Language and Environment for Statistical Computing. *R Found*. *Stat*. *Comput*. (2016).

